# Cerebral disorders in the first 7 years of life in children born post-term: a cohort study

**DOI:** 10.1186/s12887-020-1950-4

**Published:** 2020-02-03

**Authors:** Anne Hald Rolschau, Annette Wind Olesen, Carsten Obel, Jørn Olsen, Chunsen S. Wu, Poul-Erik Kofoed

**Affiliations:** 1 Department of Gynecology and Obstetrics, Lillebaelt Hospital, Kolding, Denmark; 2 Institute of Regional Health Research, University of Southern Denmark, Odense, Denmark; 3 Department of Pediatrics, Lillebaelt Hospital, Kolding Hospital, Sygehusvej 24, 6000 Kolding, Denmark; 4 Department of Gynecology and Obstetrics, Odense University Hospital, Odense, Denmark; 5 Institute of Clinical Research, University of Southern Denmark, Odense, Denmark; 6 Research Unit for Mental Public Health, Institute of Public Health, Aarhus University, Aarhus, Denmark; 7 Department of Clinical Epidemiology, Aarhus University, Aarhus, Denmark; 8 Research Unit on Gynecology and Obstetrics, Odense University Hospital, Odense, Denmark

**Keywords:** Children, Disability, Neurodevelopment, Developmental disability, Post-term delivery

## Abstract

**Background:**

To estimate the association between post-term delivery and risk of physical disabilities, mental disabilities, and seizures during the first 7 years of life.

**Methods:**

Data from 57,884 singleton infants born alive in week 39–45 by mothers included in the Danish National Birth Cohort (1997 to 2004) were analyzed, of these 51,268 were born at term (39–41 + 6) and 6616 post-term (42 + 0–44 + 6). Information on clinical endpoints was obtained from an interview at 18 months of gestational age, from a 7-year questionnaire, and from the Danish National Patient Register.

Logistic regression and Cox regression were used to estimate odds ratios and hazard rate ratios for the outcome obtained from the interview/questionnaire data and from the register-based data, respectively.

**Results:**

We found no statistically significant increased risk of physical disabilities, mental disabilities, and epilepsy among children born post-term, though for most outcomes studied a tendency towards more adverse outcomes was seen. When children born late term (week 41) were compared to children born in week 42 or later the same tendency was found.

**Conclusion:**

Post-term born children had a tendency to an excess risk of neurological disabilities as followed for up to 7 years of age.

Post-term delivery has been associated with increased perinatal mortality [1, 2] but newer studies do not support these findings, probably due to an improved management of post-term pregnancies [3, 4]. However, for unknown reasons post-term deliveries still seem to cause higher neonatal morbidity [2, 3], but it has been hypothesized to be caused by a decreased placental function in late pregnancy.

An increased risk of non-optimal motor development by increasing gestational age after 41 weeks has been reported [5, 6] and adds to the results of Moster et al. finding a higher risk of cerebral palsy in children born at 42 weeks or later compared to children born at 40 weeks [7]. The cognitive abilities of children born post-term have also been found to be negatively associated with a high gestational age at birth [8–10].

Post-term delivery has been shown to be a risk factor for seizures in the neonatal period [11] and during the first year of life [12]. Ehrenstein did, however not find evidence for an increased risk of epilepsy beyond the first year of life and until the end of the 12-years follow-up period [12], corroborating results from a Danish register-based study [4] that reported no increased risk for cerebral palsy, epilepsy, or sensorineural defects [4].

A cohort study showed that more 18 months old children born post-term achieved the assessed developmental milestones compared with children born at term (39–40 weeks) [13] and a Danish study showed a decreasing risk of impaired coordination development by increasing gestational age [14].

With respect to mental problems, a Dutch cohort study showed an increased risk of developing behavioral and emotional problems such as attention deficit hyperactivity disorder (ADHD) in children born post-term compared to children born at term when examined at 1.5 and 3 years of age [15] and an American study of children with autism spectrum disorders found a significantly higher Social Communication Questionnaire score in children born post-term (> 42 weeks) compared to children born at term, indicating a higher risk of autism spectrum disorders [16]. However, a meta-analysis of perinatal and neonatal risk factors for autism reported no association between autism and post-term birth [17].

The association between neurological complications and post-term deliveries is thus still uncertain. Therefore, further studies are needed in order to formulate evidence-based recommendations to those, who are responsible for the obstetric management of pregnancies exceeding into the post-term period. The aim of this study was to examine if post-term born children were at higher risk of physical disabilities, mental disabilities, or seizures at the age of 7 years compared to children born at term.

## Methods

Data was retrieved from the Danish National Birth Cohort (DNBC), which is a follow-up study of about 100,000 children born in the period 1997 to 2004. The DNBC is estimated to have recruited about 30% of the target population. About 50% of all general practitioners took part in the recruitment and 60% of those invited accepted the invitation [18]. The participants were interviewed by telephone twice before and twice after giving birth and asked to fill in a questionnaire when the child was 7 years old. The first and second interview at 12 and 30 weeks’ gestation, respectively, included information on reproductive, toxicological, and socio-demographic factors, lifestyle factors, and health status before and during pregnancy. The third interview at 6 months after delivery included information on health status in the last part of the pregnancy and the development of the child. The fourth interview, 18 months after delivery, mainly added information on development and milestones of the child. In the questionnaire at 7 years of age information on developmental milestones, health status, physical skills, and eating habits were included (www.DNBC.dk).

Data from the interviews and the 7-year questionnaire were linked to data from the Danish National Patient Register.

**Fig. 1:**
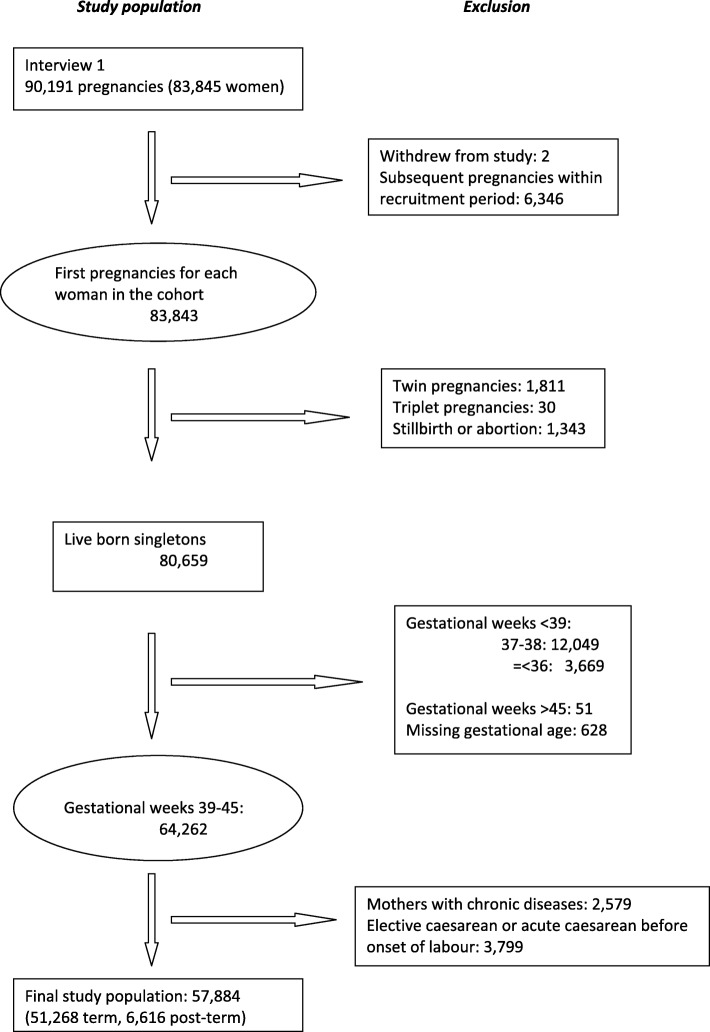
Study population. Flow chart showing number of pregnancies and number of live born children included in the study

Data included in this study was obtained from a cohort of 90,191 pregnancies where the mothers participated in the first pregnancy interview. Two women withdrew their informed consent to participate in the study. Subsequent pregnancies during the inclusion period, multiple gestations, stillbirth, abortion, and deliveries below gestational age 39 + 0 weeks or above 45 + 0 weeks were excluded. We identified 64,262 children born in gestational week 39–45 (17,112 born in week 39, 23,313 in week 40, 16,531 in week 41, 6985 in week 42, 299 in week 43 and 22 in week 44). Of these, 2579 children born of mothers who reported chronic diseases [essential hypertension (O100-O109, O130-O139, I100-I109), preeclampsia (O140-O149), thyroid disease (O992-O992a), diabetes type 1 (E100-E109), diabetes type 2 (E110-E119A), gestational diabetes (O240-O249)] according to the 10th revision codes of the International Statistical Classification of Diseases and Related Health Problems (ICD10) were excluded. Finally, 3799 cases of elective caesarean section (O820) or acute caesarean section before spontaneous labor (O821A) both at-term and post-term were excluded. Thus infants born after a caesarian section were only included if the caesarian was decided during delivery. This left 57,884 children for the final analysis (Fig. 1).

Data on gestational age was retrieved from the Danish National Patient Register. In the study period, the gestational age was estimated by ultrasound and used if the last menstrual period was considered unreliable [19]. Post-term delivery was defined as a pregnancy with delivery in gestational week 42 + 0 to 44 + 6, and term delivery as delivery in gestational week 39 + 0 to 41 + 6 (reference group).

### Outcome measures

Outcomes of interest were extracted from the interview/questionnaire obtained from the DNBC, and the Danish National Patient Register. Background and baseline information concerning the maternal health status and the pregnancy were obtained from the first interviews in the DNBC. The interview/questionnaire outcomes concerning the child were obtained from the DNBC including information on 1) physical disabilities (chronic disease or handicap from the 7-year questionnaire, and developmental deficit from the 18-months interview), 2) seizures (epilepsy and febrile seizures from the 7-year questionnaire), and 3) mental disabilities (speech development, late start at school, and psychiatric case status from the 7-year questionnaire).

For assessing psychiatric problems an extended version of the Strengths and Difficulties Questionnaire (SDQ) [20–22] was used to provide a measure of the overall status of behavioral or social difficulties. We used the predictive algorithme based on one informant to define psychiatric caseness. The algorithme is based on the 20 questions for the subscales: 1) emotional symptoms (5 items), 2) conduct problems (5 items), 3) hyperactivity/inattention (5 items), and 4) peer relationship problems (5 items), and the added score was combined with the score of daily impact. Based on this definition about 8% of the population was identified as having a possible psychiatric disorder.

Most of the answers to the remaining questions in the interview and the questionnaire were truncated as “yes”- or “no”-answers, i.e. “Has a doctor ever said that your child had epilepsy?”

Diagnoses based on the Danish version of the ICD10 were obtained from the Danish National Patient Register as recorded during the entire follow-up period. The diagnoses included physical disabilities as cerebral and other palsies (DG80.0–9, DG81.0–9, DG82.0–5, DG83.0–9), seizures as epilepsy (DG40.0–9, DG41.0–9) and febrile seizures (DR560, DR560A) and mental and psychiatric disabilities (DF40.0–9, DF42.0–9, DF80.0-DF83.9, DF90.0-DF98.9), including infantile autism (F84.0-F89.9).

### Statistical analysis

Differences in characteristics between term and post-term were compared using chi square test for categorical variables.

The children were followed from birth until the time of first recorded occurrence of the outcomes of interest (physical disabilities, mental disabilities, and seizures), emigration, death, or end of follow-up, whichever came first. The underlying time scale was age of the child in days. We used Cox proportional hazards models to estimate hazard rate ratios (HRs) with 95% confidence intervals (95% CI) for the onset of the outcomes registered in the Danish National Patient Register.

The analyses were conducted for each outcome, respectively. The results were adjusted for potential confounders including maternal age (continuous variable), parity (0/1+), socio-occupational status (high, medium, and low), smoking (yes/no but partner smoking/no), pre-pregnancy body mass index (continuous variable), and child’s sex (boy/girl).

The Cox proportional hazards assumptions were examined using log-log plots, which showed no severe violation of proportionality.

We conducted both crude as well as adjusted logistic regression to estimate odd ratios (ORs) with 95% confidence intervals for data from DNBC.

Finally, in a sub-analysis, the outcome mesurements were estimated for children born post-term (42 + 0–44 + 6, 6616 children) compared to late term (41 + 0–41 + 6, 15,390 children) (as reference).

Statistical analyses were performed using STATA version 13 (Stata Corp., College Station, Texas, USA).

## Results

Figure 1 illustrates how the 57,884 children participating in the final analysis were identified. In Table 1, maternal characteristics are shown. The group of women who delivered post-term more often gave birth to boys and was characterized by more nulliparas, and with higher pre-pregnancy body-mass-index. There were slightly fewer with fever during pregnancy. We found no significant differences in socio-occupational status, maternal age, or the frequency of maternal epilepsy, psychiatric disorder, folic acid consumption, smoking and alcohol consumption (Table 1). Very few admitted using hash (137/48,345 (0.3%) vs. 16/6279 (0.3%)) or other drugs during pregnancy (20/48,354 (0.04%) vs. 3/6281 (0.05%)).

**Table 1:** Characteristics of 57,884 women who delivered at term (39 + 0–41 + 6 gestational weeks) or post-term (42 + 0–44 + 6 gestational weeks)

	At term		Post-term	
	n	%	N	%
Total	51,268	100.0	6616	100.0
Maternal age				
10–25	8846	17.3	1159	17.5
26–30	22,360	43.6	2862	43.3
31–35	15,696	30.6	2004	30.3
36–50	4366	8.5	591	8.9
				*P* = 0.616
Parity				
0	24,596	48.0	3670	55.5
1	18,469	36.0	1971	29.8
2	6720	13.1	796	12.0
3	1217	2.4	146	2.2
4+	238	0.5	29	0.4
Missing	28	0.1	4	0.1
				*P* = 0.000
Pre-pregnancy BMI				
< 18.5	2294	4.5	219	3.3
18.5 - < 25	35,359	69.0	4201	63.5
25 - < 30	9329	18.2	1442	21.8
> 30	3474	6.8	627	9.5
Missing	812	1.6	127	1.9
				P = 0.000
Child’s sex				
Male	25,788	50.3	3484	52.7
Female	25,479	49.7	3132	47.3
Missing	1	0.0	0	0.0
				*P* = 0.001
Socio-occupational status				
High	27,109	52.9	3511	53.1
Medium	19,206	37.5	2478	37.5
Low	4764	9.3	606	9.2
Missing	189	0.4	21	0.3
				*P* = 0.902
Smoking				
Yes	7423	14.5	927	14.0
No, but partner smoking	7547	14.7	1057	16.0
No	24,839	48.4	3165	47.8
Missing	11,459	22.4	1467	22.2
				*P* = 0.054
	39 + 0–41 + 6		42 + 0–44 + 6	
	n	%	N	%
Maternal epilepsy, medicated				
Yes	113	0.2	11	0.2
No	39,723	77.5	5140	77.7
Missing	11,432	22.3	1465	22.1
				*P* = 0.638
Psychiatric disorder				
Yes	3869	7.5	467	7.1
No	47,357	92.4	6144	92.9
Missing	42	0.1	5	0.1
				*P* = 0.364
Alcohol consumption during pregnancy				
Yes	9351	18.2	1199	18.1
No	30,393	59.3	3939	59.5
Missing	11,524	22.5	1478	22.3
				*P* = 0.924
Fever during pregnancy				
Yes	11,138	21.7	1390	21.0
No	36,898	72.0	4848	73.3
Missing	3232	6.3	378	5.7
				*P* = 0.049
Folic acid consumption during pregnancy				
Yes	12,070	23.5	1567	23.7
No	36,223	70.7	4707	71.1
Missing	175	0.3	12	0.2
				*P* = 0.062

No statistically significant differences were found between children born at term (gestational age 39 + 0 to 41 + 6) and the post-term born children (42 + 0 to 44 + 6) in risk of physical disabilities or seizures when analyzing the mother’s answers at the 18-month interview and the 7-year questionnaire. An increased risk of psychiatric caseness as evaluated by the SDQ questionnaire was seen in the post-term group (Table 2). When using data from the Danish National Patient Register no statistically significant differences were found for cerebral palsy, psychiatric diagnoses or epilepsy, whereas more in the post-term group got febrile seizures (Table 3). However, for most conditions, apart from epilepsy and psychiatric diagnoses when analyzed on data from the Danish National Patient Register, a tendency towards an increased risk was seen for the post-term group.

**Table 2:** Physical disabilities, mental disabilities, and seizures in children born post-term compared to children born at term. Data from the 7-year interview (DNBC)

Variable	Logistic regression OR	Adjusted logistic regression^a^ OR (95% CI)	n_1_/N_1_^b^	n_2_/N_2_^c^
Developmental deficit^d^	1.09	1.12 (0.88–1.43)	542/50,239	76/6465
Chronic disease or handicap	1.14	1.10 (0.97–1.25)	1937/50,027	278/6424
Speech development	1.12	1.11 (0.99–1.25)	2667/30,619	374/3898
Late start at school	1.03	0.99 (0.74–1.33)	383 /30,687	52/3905
Case-caseness SDQ	1.18	1.14 (1.02–1.28)	2494/30,750	372/3914
Epilepsy	1.09	1.14 (0.72–1.80)	146/29,994	21/3826
Medication for epilepsy	1.36	1.38(0.80–2.40)	105/2041	16/228
Febrile seizures	1.07	1.02 (0.90–1.15)	2430/43,437	323/5574

*OR* Odds ratio calculated by logistic regression analysis

^a^ adjusted for maternal age, parity, pre-pregnancy BMI, child’s sex, and socio-occupational status

^b^ number (n_1_) of cases amongst children born at term (N_1_) in adjusted logistic regression analysis

^c^ number (n_2_) of cases amongst children born post-term (N_2_) in adjusted logistic regression analysis

^d^ from 18 months interview

**Table 3:** Physical disabilities, mental disabilities, and seizures in children born post-term compared to children born at term. Data from the Danish National Patient Register

Variable	Cox regression HR	Adjusted cox regression^a^ HR (95% CI)	n_1_/N_1_^b^	n_2_/N_2_^c^
Cerebral and other palsies	1.15	1.09 (0.63–1.87)	106/50,021	15/6437
Psychiatric diagnoses	0.52	0,51 (0.26–1.00)	128/50,021	9/6437
Autism	–	–	4/51,268	0/6616
Epilepsy	0.28	0.30 (0.04–2.20)	28/47,970	1/6248
Febrile seizures	1.56	1.54 (1.12–2.12)	226/50,245	46/6466

*HR* Hazard ratio calculated by cox regression analysis

^a^ adjusted for maternal age, parity, pre-pregnancy BMI, child’s sex and socio-occupational status

^b^ number (n_1_) of cases amongst children born at term (N_1_)

^c^ number (n_2_) of cases amongst children born post-term (N_2_)

In a sub-analysis comparing the post-term group (42 + 0–44 + 6) (as exposed) with the late term group (41 + 0–41 + 6) (as reference), we found an increased risk of developmental deficit (OR 1,33; 95%-CI: 1.00–1.77), chronic disease or handicap (OR 1,16; 95%-CI: 1.00–1.35), and psychiatric caseness (OR 1.19; 95%-CI: 1.04–1.36) in interview/questionnaire data from DNBC and a significantly increased risk of febrile seizures (HR 1.60; 95%-CI: 1.09–2.33) using data from the Danish National Patient Register (Tables 4 and 5).

**Table 4:** Physical disabilities, mental disabilities, and seizures in children born post-term in week 42 or later compared to that of children born in week 41. Data from the 7-year interview (DNBC)

Variable	Logistic regression OR	Adjusted logistic regression^a^ OR (95% CI)	n_1_/N_1_^b^	n_2_/N_2_^c^
Developmental deficit^d^	1.31	1.33 (1.00–1.77)	138/15,086	76/6465
Chronic disease or handicap	1.21	1.16 (1.00–1.35)	551/15,010	278/6424
Speech development	1.14	1.12 (0.98–1.28)	794/9169	374/3898
Late start at school	1.06	1.00 (0.72–1.40)	113/9149	52/3890
Psychiatric caseness SDQ	1.23	1.19 (1.04–1.36)	725/9204	372/3914
Epilepsy	1.09	1.17 (0.69–1.99)	43/8934	21/3811
Medication for epilepsy	1.51	1.51(0.80–2.87)	29/610	16/228
Febrile seizures	1.05	1.00 (0.87–1.14)	739/13,023	323/5574

*OR* Odds ratio calculated by logistic regression analysis

^a^ adjusted for maternal age, parity, pre-pregnancy BMI, child’s sex and socio-occupational status

^b^ number (n_1_) of cases amongst children born 41 + 0–41 + 6 (N_1_) in adjusted logistic regression analysis

^c^ number (n_2_) of cases amongst children born 42 + 0–44 + 6 (N_2_) in adjusted logistic regression analysis

^d^ from 18 months interview

**Table 5:** Physical disabilities, mental disabilities, and seizures in children born post-term in week 42 or later compared to that of children born in week 41. From the Danish National Patient Register

Variable	Cox regression HR	Adjusted cox regression^a^ HR (95% CI)	n_1_/N_1_^b^	n_2_/N_2_^c^
Cerebral and other palsies	1.77	1.66(0.85–3.22)	21/14,428	15/6219
Psychiatric diagnoses	0.58	0,60(0.29–1.25)	34/15,023	9/6437
Autism			1/48	0/0
Epilepsy	0.29	0.29 (0.04–2.29)	8/12,649	1/5495
Febrile seizures	1.59	1.60 (1.09–2.33)	66/15,086	46/6466

*HR* Hazard ratio calculated by cox regression analysis

^a^ adjusted for maternal age, parity, pre-pregnancy BMI, child’s sex and socio-occupational status

^b^ number (n_1_) of cases amongst children born 41 + 0–41 + 6(N_1_)

^c^ number (n_2_) of cases amongst children born 42 + 0–44 + 6 (N_2_)

## Discussion

Neurodevelopmental outcome of 51,268 infants born at term and 6616 born post-term were examined using data from DNBC and the Danish National Patient Register. No statistically significant increased risk of physical or mental disabilities was found in post-term born infants as compared to infants born at-term. For most of the conditions examined a tendency towards an increased risk was seen (Tables 2 and 3).

A discrepancy in the results for febrile seizures was found as the mothers up to ten times more often stated that the child had experienced a febrile seizure than that reported in the Danish National Patient Register which may be due to underreporting in the national register (0.5%, 272/56,711) as compared to self-reporting (5.6%, 2753/49,011), as the incidence of febrile seizures in Denmark is approximately 4% [23]. Very few children had psychiatric diagnoses, whereas using the SDQ questionnaire 8.1% in the term group and 9.5% in the post-term group had a score indicating a psychiatric disorder, demonstrating that only the more severe cases had been diagnosed and thus reported to the Danish National Patient Register, whereas the SDQ is a screening for psychiatric and mental challenges covering more aspects of the psychiatric and mental wellbeing of the child.

We compared the outcome of post-term children with that of children born at late term and found a higher risk of adverse outcomes supporting the present recommendation in Denmark for induction of births at gestational age 41 + 3, and thus giving birth before week 42 + 0.

Most women who delivered post-term were healthy and had pregnancies without complications. The effect on the infant of being born post-term could at least in part be due to the increased exposure to the intrauterine environment. Indication for induction in this cohort was a prolonged pregnancy reducing the risk of selection bias and confounding by indication.

The major limitation of the study is in the nature of observational research where unknown confounders may have masked or exaggerated differences, why there is a possibility of residual confounding. Misclassification of gestational age could potentially bias the results in both directions, but it is less likely in this data set with good quality gestational age data.

We had a large study population with close follow up. However, the low incidence of some of the studied outcomes limited our power. On the other hand, the large study-population also revealed even small to moderate differences with limited clinical relevance.

We excluded mothers with chronic diseases as their deliveries were often induced at term and could confound our results. Acute caesareans instituted before the onset of spontaneous labor are mostly due to maternal factors and were therefore censured as were all elective caesareans in order to compare the outcome of infants born spontaneously at term with infants born after pregnancies allowed to progress post-term. The results were adjusted for confounding by known risk factors of post-term delivery [24, 25].

However, ideally an unbiased study on the health consequences for the child of being born post term would require a randomized controlled trial set up. Pregnant women reaching term could be randomized to active or passive follow up. As such a study would hardly be acceptable we study interventions done by clinicians which make us susceptible to ‘confounding by indication’. Those who have their pregnancy induced may have more risk factors than those who are allowed to proceed to become post-term.

The DNBC has previously been shown not to have significant selection bias for severe outcomes [26]. The combination with the Danish National Patient Register furthermore reduced the risk of bias due to differential loss to follow up, which can be a problem in a prospective cohort that does not include an entire population [27].

Information on gestational age was obtained from the Medical Birth Register, and usually based on ultrasound examination. The precision of gestational age after introduction of routine ultrasound scans in Denmark is high, and even the last menstrual period based gestational age is often valid [25]. Routine ultrasound scans were used from the early 1990’ies in case of unreliable last menstrual period, and the frequency of routine ultrasound screening for malformations increased from 40 to 54% from 1989 to 90 to 1994–95, both increasing the validity of the data on gestational age at birth [19].

### Interpretation

The analysis of several different variables increased the risk of finding significant associations due to chance and replication studies are thus needed. We found neither statistically significant adverse nor protective long-term effects on the studied outcomes when comparing post-term born children with all children born at term or with children born in week 41. However, both compared to all children born at term and children born in week 41 a non-significant tendency towards an adverse effect was seen for most studied outcomes. This corroborates the findings of a large study on the association between school performance at the age of 16 years and gestational age reporting a decreased performance for children born post-term after week 41 [28], and of a study finding increased intellectual disabilities with increasing gestational age post-term from week 42, an association persisting in a cohort of matched siblings suggesting that they were robust against confounding by shared familial traits [29].

## Conclusion

Post-term delivery is considered an independent risk factor for neonatal morbidity [3], but long-term neurodevelopmental consequences have been less well studied. In this large observational study, a tendency to an increased risk of the studied outcomes in post-term children was found following the obstetric practice in Denmark at that time.

## Data Availability

The data that support the findings of this study are available from Bedre Sundhed i Generationer (BSIG.dk) but restrictions apply to the availability of these data, which were used under license for the current study, and so are not publicly available. Data are however available from the authors upon reasonable request and with permission of Bedre Sundhed i Generationer (BSIG.dk).

## Abbreviations

ADHD: Attention deficit hyperactivity disorder
DNBC: Danish National Birth Cohort
ICD10: The International Statistical Classification of Diseases and Related Health Problems 10th Revision
SDQ: Strengths and Difficulties Questionnaire
